# Editorial: Beneficial microbe-plant interactions under biotic/abiotic stress conditions

**DOI:** 10.3389/fmicb.2023.1294042

**Published:** 2023-10-11

**Authors:** Lin Chen, Yunpeng Liu, Zhihui Xu, Yang Song

**Affiliations:** ^1^National Permanent Scientific Research Base for Warm Temperate Zone Forestry of Jiulong Mountain, Experimental Center of Forestry in North China, Chinese Academy of Forestry, Beijing, China; ^2^State Key Laboratory of Efficient Utilization of Arid and Semi-arid Arable Land in Northern China, The Institute of Agricultural Resources and Regional Planning, Chinese Academy of Agricultural Sciences, Beijing, China; ^3^Jiangsu Provincial Key Lab of Solid Organic Waste Utilization, Jiangsu Collaborative Innovation Center of Solid Organic Wastes, Nanjing Agricultural University, Nanjing, China; ^4^Plant-Microbe Interactions, Department of Biology, Science4Life, Utrecht University, Utrecht, Netherlands

**Keywords:** plant-associated microbe, biotic/abiotic stress, rhizosphere, endophytic, plant-microbe interactions, plant resistance, plant tolerance

Plants are facing a range of biotic and abiotic stressors, including pests, pathogens, salinity, drought, cold, heavy metal stress, and nutrient deficiencies. These challenges restrict agroforestry production. The plant-associated microbiome constitutes the second genome of plants and has garnered significant attention due to its pivotal roles in plant growth, productivity, and health (Trivedi et al., 2020). Both the composition and function of the microbiome are influenced by plant-microbe interactions and environmental factors, such as biotic and abiotic stress conditions. However, the limited understanding of the interplay between beneficial microbes and plants hampers the utilization of plant-associated microbes for sustainable agriculture and forestry ecosystems under stress conditions. This Research Topic aims to deepen our understanding of the functions and response mechanisms of beneficial microbes and plants to biotic and abiotic stressors.

This Research Topic comprises 22 articles (21 research articles and one review article) that explore the interactions between beneficial microbes and plants under biotic or abiotic stress conditions. The plant-associated beneficial microbes include rhizosphere and endophytic microbes. These articles illuminate the roles of beneficial microbes in plant health, identify novel beneficial strains, describe the characteristics of rhizosphere or endophytic microbial communities across varying environments and plant genotypes, and elucidate signaling mechanisms governing the interactions between microbes and plants under stress conditions, thereby contributing to the potential application of beneficial microbes in future sustainable food, fuel, and fiber crop production.

## Biotic stress

Numerous studies have reported that certain plant-associated microbes can enhance plant resistance to diseases. Some beneficial microbes can induce systemic resistance against phytopathogens in plants without direct contact with pathogens (Pieterse et al., 2014). Other beneficial microbes exert their protective effects by directly producing microbial metabolites that antagonize pathogens. For foliar diseases, direct spraying of beneficial endophytic strains onto the affected areas of the plant has proven effective for disease control. For instance, the endophytic *Bacillus amyloliquefaciens* KRS005 activates plant defense responses and inhibits the morphological development of *Botrytis cinerea* mycelia, thereby controlling gray mold when sprayed on leaves (Qi et al.). *Paraburkholderia phytofirmans* PsJN also effectively controls grape gray mold disease when directly sprayed on inflorescences rather than through root inoculation. PsJN can be attracted by *B. cinerea* and inhibit its spore germination (Miotto Vilanova et al.). Secreted metabolites and volatiles of endophytic *Microbacterium testaceum* mediate antagonistic effects on the pathogen *Magnaporthe oryzae*, thus playing a significant role in controlling rice blast disease when applied via spraying (Patel et al.).

For soil-borne diseases, root inoculation with beneficial rhizosphere microbes has shown efficacy. Wilt disease is a kind of soil-borne disease caused by different fungal pathogens, such as *Verticillium* and *Fusarium*, which has caused significant economic losses in agriculture and forestry industries. Kong et al. demonstrated that the volatile organic compounds (VOCs) produced by *Trichoderma koningiopsis* T2 can inhibit the formation of microsclerotia, suppress activities of certain cell wall-degrading enzymes, and downregulate genes associated with melanin synthesis in the pathogen *Verticillium dahliae*. These mechanisms were thought to contribute to the biocontrol efficacy of T2 against *Verticillium* wilt disease. Besides direct antagonism against pathogens, microbial agents can reshape the soil microbiome by changing soil properties and metabolites and enriching potential beneficial microbes, thereby aiding plants in combating soil-borne diseases (Li X. et al.; Wang et al.). Appropriate farming methods can also mitigate the occurrence of such diseases. For example, maize-soybean intercropping has been shown to suppress Fusarium wilt disease in soybeans through reshaping the rhizosphere bacterial community and recruiting more beneficial bacteria (Chang et al.).

In addition to the biocontrol of pathogen infections, plant-associated microbes also play crucial roles in plant resistance to insect attack. Li G. et al. reported distinct microbiome diversity in plants and soil between insect-resistant and insect-susceptible plants. The origin of insect microbiota is mainly from plant stems and partially from soil. After insect attack, the microbiome in insect-susceptible plants and rhizosphere soil shifts toward that of insect-resistant plants. This study reveals the role of dynamic changes of microorganisms within plants and insects in determining biocontrol agents to kill insects upon direct contact and can also enhance plant growth and indirectly suppress spider mite populations on tomatoes (Rasool et al.).

## Abiotic stress

A multitude of studies have investigated the roles of beneficial microbes in enhancing plant tolerance to abiotic stress, such as salinity. Halophytes, for example, can recruit salt-resistant microorganisms to their rhizosphere to enhance their salt tolerance. Bacterial secretory systems play important roles in environmental adaptation. For instance, many plant symbiotic Gram-negative bacteria contain the type VI secretion system (T6SS) that enhances bacterial competitiveness and biofilm formation (Peng et al.). Some beneficial bacteria trigger systemic salt tolerance in plants through producing VOCs (Luo et al.). Inoculation with certain beneficial strains alters the root endophytic microbial community, thereby enhancing plant salt stress tolerance (Xu et al.; Zeng et al.). Arbuscular mycorrhizal fungi (AMF) alleviate high salt concentration stress in *Xanthoceras sorbifolium* through reducing Na^+^ content, improving osmotic tolerance and antioxidant activity in plants (Zong et al.). Some dark septate endophytes (DSEs), belonging to Ascomycota with high melanin-producing activities, can also enhance plant salt tolerance. However, the melanin in DSEs contributes to the colonization progress but not salt tolerance of the plant (Gaber et al.).

Besides salt stress, beneficial microbes can trigger plant tolerance to other abiotic stresses, such as drought, cold and chromium (Cr) stress. Melatonin has been observed to improve the colonization of AMF. Furthermore, melatonin can amplify the cold stress tolerance in perennial ryegrass induced by AMF through accumulating protective molecules and enhancing antioxidant activity (Wei et al.). Fan et al. discovered that drought-induced rhizosphere bacterial communities play a key role in improving alfalfa tolerance to drought stress. The drought-resistant bacteria are abundant in the rhizosphere of drought-tolerant plant varieties under normal conditions and are only recruited to the rhizosphere of drought-sensitive plant varieties following exposure to drought stress. The composition of plant-associated microbes varies depending on both plant varieties and environmental conditions (Zheng et al.). Mao et al. revealed that the enhanced dominant bacteria in the rhizosphere of *Canna indica* under Cr stress are associated with the altered metabolites secreted by roots.

Some nutrients are important for plants and are often poorly available in soil. However, their availability can be affected by microbial activity. Plant response to beneficial microbes is contingent on nutrient concentrations, their availabilities, and the specific identities of these beneficial microbes. For instance, some beneficial strains can enhance phosphorus (P) content in plants under P-deficient conditions due to their phosphate-solubilizing ability (Chen et al.). Orellana et al. found that some proteobacterial strains reduce iron (Fe) content in plants, possibly by downregulating iron uptake-related genes in plants. Meanwhile, the rhizosphere microbial communities are also impacted by different nutrient supply conditions. High nitrogen addition inhibits the stability and diversity of rhizosphere bacterial communities, whereas low nitrogen addition enriches ammonia-oxidizing bacteria to improve nitrogen use efficiency (Li Y. et al.). This finding can provide guidance for the use of nitrogen fertilizers.

In summary, all contributions collected in this Research Topic highlight the significance of plant-associated beneficial microbes in countering both biotic and abiotic stress conditions. It illuminates the intricate mechanisms of plant-microbe interactions under these challenging conditions and their applications in sustainable agricultural and forestry ecosystems. Further studies are necessary to fully grasp the potential of beneficial microbes in enhancing plant growth and combating various stresses.

## Author contributions

LC: Writing—original draft, Writing—review and editing. YL: Writing—original draft, Writing—review and editing. ZX: Writing—review and editing. YS: Writing—review and editing.
